# Clinical Use of a Multivariate Electroencephalogram (Narcotrend) for Assessment of Anesthetic Depth in Horses during Isoflurane–Xylazine Anesthesia

**DOI:** 10.3389/fvets.2016.00025

**Published:** 2016-03-17

**Authors:** Julia Tünsmeyer, Klaus Hopster, Sabine B. Kästner

**Affiliations:** ^1^Small Animal Clinic, University of Veterinary Medicine Hannover Foundation, Hannover, Germany; ^2^Equine Clinic, University of Veterinary Medicine Hannover Foundation, Hannover, Germany

**Keywords:** horses, EEG, Narcotrend, anesthesia, isoflurane

## Abstract

**Objective:**

To investigate the use of the Narcotrend electroencephalogram (EEG) monitor for the assessment of anesthetic depth in horses undergoing xylazine balanced isoflurane anesthesia.

**Study design:**

Blinded experimental study.

**Animals:**

Seven healthy warm-blooded horses, aged 10.6 ± 5.9 years, weighing 535 ± 55 kg.

**Methods:**

Horses were anesthetized for a terminal surgical trial with xylazine, thiopentone, and guaiphenesin for induction and isoflurane and xylazine continuous rate infusion for maintenance. After surgery, an EEG was recorded and processed by the Narcotrend monitor. It displays an index [Narcotrend index (NI)] between 0 and 100, which is supposed to indicate anesthetic depth. This index was recorded and correlated with eight different end tidal (ET) isoflurane concentrations between 0.8 and 2.2 vol%. In addition, anesthetic depth was numerically scored based on common clinical signs with a score of 1 (plane “too deep”) to 4 (plane “too light”). After testing for normal distribution, both clinical scores and NI were correlated with different ET isoflurane concentrations using Spearman rank correlation.

**Results:**

Correlation of NI with ET isoflurane concentrations was poor (*r*_s_ = 0.24). The NI ranged between maximal 48 and minimal 13 in the horses. The clinical scores decreased with increasing ET isoflurane concentrations (*r*_s_ = 0.80). They ranged from 1 to 4 in different horses at the concentrations investigated.

**Conclusion:**

In this study, the NI did not seem to be useful for assessment of anesthetic depth in horses receiving isoflurane anesthesia balanced with a xylazine constant rate infusion.

## Introduction

The exact determination of anesthetic depth is still a challenge, and the electroencephalogram (EEG) has been extensively investigated as a tool to monitor anesthetic depth in human beings and also in horses. Precise assessment of central nervous system (CNS) activity in horses is of particular importance because too light anesthetic planes may be associated with movement, leading to injury of the horse or personnel. Too deep anesthetic planes increase the risk of complications, such as cardiorespiratory depression and postanesthetic myopathy (1, 2). This might be one reason for the high perioperative mortality rate in this species compared to small animals (3, 4).

Different studies investigated EEG power spectrum analysis in horses (5, 6). However, in human medicine, this technique has more and more been replaced by the use of multivariate monitors using algorithms, such as the bispectral index (BIS). In humans, BIS seems to be a reliable indicator of anesthetic depth with different anesthetic protocols (7). Similarly, in veterinary medicine, BIS was inversely correlated with inhalation anesthetic concentrations in dogs, cats, and pigs (8–10). However, in isoflurane-anesthetized horses, BIS did not precisely indicate the degree of CNS depression (11).

The Narcotrend is a different multivariate EEG monitor, which uses algorithms based on a visual classification of the human sleep EEG, classical spectral parameters, and algorithms for recognition of suppression lines (12). In the same way as BIS, it displays anesthetic depth by a dimensionless index between 0 (electrical silence) and 100 (awake). Multiple human clinical and validation studies investigating the Narcotrend are available, including comparison of Narcotrend to BIS, with good results (13, 14). Nevertheless, veterinary experience is limited (15, 16). In dogs anesthetized with inhalant agents, Narcotrend index (NI) differed between anesthetic planes assessed clinically as “too deep” and “adequate,” but not between planes assessed as “too light” and “adequate” (15).

To the best of our knowledge, no published data are available about the Narcotrend assessing anesthetic depth in horses. Therefore, the aim of this study was to investigate if the NI correlates with different end tidal (ET) isoflurane concentrations in horses and to compare this correlation to common clinical signs that are routinely used to monitor anesthetic depth in horses.

We hypothesized that the NI would be able to detect different stages of anesthesia reflected by different ET isoflurane concentrations.

## Materials and Methods

Seven warm-blooded horses, aged 10.6 ± 5.9 years, weighing 535 ± 55 kg, were anesthetized for a terminal experimental abdominal surgery approved by the local animal welfare committee (number 33.14-42502-04-11/0572). The EEG part of the study was started after termination of the abdominal surgery, because it was not possible to vary anesthetic depth during the surgical study. The surgeries lasted approximately 2 h. After investigating the NI at different ET isoflurane concentrations, the horses were euthanized with 80 mg kg^−1^ pentobarbital, and the cadavers were used for anatomical studies.

Prior to anesthesia, all the horses were assessed to be healthy by physical examination and complete blood cell count (American Society of Anesthesiologists classification I/II). All the horses were premedicated intravenously (IV) with xylazine (Xylapan, Vetoquinol, Germany) until deeply sedated (0.8–1.1 mg kg^−1^) and placed in stocks with a swinging door. Guaiphenesin (Myolaxin15%, Vetoquinol, Germany) was infused IV to effect (75–100 mg kg^−1^) until horses became ataxic, and anesthesia was induced with 5 mg kg^−1^ thiopental IV (Trapanal, Nycomed Deutschland GmbH, Germany). Following orotracheal intubation (Cuffed endotracheal silicone tube, Smiths Medical ASD Inc., Germany; ID 25–30 mm), horses were hoisted on the surgical table, placed in dorsal recumbency, and connected to a large animal circle system. Anesthesia was maintained with isoflurane (Isofluran CP, CP-Pharma, Germany) delivered in 4–8 l min^−1^ of 100% oxygen (O_2_), and a xylazine infusion at a continuous rate of 0.75 mg kg^−1^ h^−1^ delivered by a syringe driver (Braun Perfusor Compact S, B. Braun Melsungen AG, Germany), which was started immediately after induction.

Lactated Ringer’s solution (Ringer-Laktat-Lösung, B. Braun Melsungen AG, Germany) was administered at a rate of 10 ml kg^−1^ h^−1^, and dobutamine (Dobutamin-ratiopharm 250 mg, ratiopharm GmbH, Germany) (0.5 μg kg^−1^ min^−1^) was administered *via* syringe driver (Braun Perfusor Compact S, B. Braun Melsungen AG, Germany) to maintain mean arterial blood pressure (MAP) above 60 mmHg. A MAP below 60 mmHg was defined as hypotension. For the evaluation of anesthetic depth, a MAP >90 mmHg was presumed as “elevated,” a MAP <90 and >70 mmHg as “normal,” a MAP <70 and >65 mmHg as “minimally depressed,” a MAP <65 and >60 mmHg as “moderately depressed,” and a MAP <60 mmHg as “markedly depressed” (Table 1).

**Table 1:** **Clinical scoring system of anesthesia depth in horses [modified from Hubbell and Muir (17)]**.

Anesthetic plane	Clinical score	Pupil position/size	Eye reflex (P, palpebral; C, corneal)	Heart rate/blood pressure/(spontaneous respiration)
Too light	4	Central/large or small	P: active; C: active	Normal or elevated/breathing against ventilator; occasional swallowing
Medium	3	Ventromedial/small or medium	P: depressed; C: mildly depressed	Normal or minimally depressed
Medium–deep	2	Central/medium	P: depressed; C: depressed	Minimally to moderately depressed
Too deep	1	Central/large	P: absent; C: markedly depressed	Markedly depressed

*A mean arterial pressure (MAP) >90 mmHg was presumed as “elevated,” a MAP ≤90 and ≥70 mmHg as “normal,” a MAP <70 and ≥65 mmHg as “minimally depressed,” a MAP <65 and >60 mmHg as “moderately depressed,” and a MAP ≤60 mmHg as “markedly depressed”*.

*A heart rate (HR) >45 beats per minute (bpm) was supposed to be elevated, a HR between 30 and 45 bpm was thought to be normal, and a HR <30 bpm was thought to be depressed*.

Pressure-cycled intermittent positive pressure ventilation (Bird Mark 7, Vet.-Tec. Model JAVC 2000 J.D. Medical Distributing Company, Phoenix, AZ, USA) was initiated immediately after connection to the anesthetic machine in all horses and adjusted to maintain end tidal carbon dioxide (ET CO_2_) between 4.7 and 6 kPa (35–45 mmHg). The transverse facial artery was cannulated with a 21-G catheter (Introcan-W, B. Braun Melsungen AG, Germany) for invasive blood pressure monitoring and arterial blood sampling. The catheter was connected to a pre-calibrated pressure transducer (Gould Statham Transducer, PD 23 ID, USA) *via* fluid-filled, non-compliant extension lines, and the pressure transducer was positioned at the level of the shoulder joint and zeroed to atmospheric pressure. Respiratory gases were sampled continuously from the *y*-piece. Electrocardiogram (ECG), respiratory rate, inspiratory and ET CO_2_, O_2_ and isoflurane concentrations, O_2_ saturation of hemoglobin *via* pulse oximetry (SpO_2_), and arterial blood pressure were monitored continuously with a multiparametric anesthesia monitor (Kardiocap 5 monitor, Datex-Ohmeda GmbH, Germany) and recorded every 10 min. Arterial blood gases were sampled every 30 min and immediately analyzed (AVL995, AVL Medizintechnik, Germany).

The Narcotrend monitor (Monitor Technik, Bad Bramstedt, Germany) was used for recording and processing of the raw EEG. The raw EEG signal was recorded by standard silver ECG needle electrodes using a single-channel registration. The recording electrodes were subcutaneously placed modified from Mayhew and Washbourne (18), with the active electrodes bilaterally placed over the zygomatic processes and the reference placed caudal to the poll. Correct needle placement was checked by automatic impedance measurements of the monitor. The positions of needles were changed when the impedance was 6 kΩ or higher. The EEG was continuously recorded and processed to the Narcotrend stages and indices, and the data were stored by the monitor for off-line analysis.

The Narcotrend monitor analyzes the EEG signal in epochs of 20 s, and the display is updated every 5 s. The monitor displays an anesthetic stage between A (awake) and F (very deep hypnosis), which is further subdivided in the NI ranging between 100 (awake) and 0 (isoelectricity). Due to greater precision and better comparability to other EEG monitors, such as the BIS, only the NI was analyzed in this study.

Randomly assigned by drawing labeled papers from a box to either an ascending or a descending order, vaporizer settings were adjusted to achieve eight different ET isoflurane concentrations (0.8, 1.0, 1.2, 1.4, 1.6, 1.8, 2.0, and 2.2 vol%). The observer assessing anesthetic depth by clinical signs was blinded to the vaporizer settings and the EEG monitor readings. ET concentrations were kept constant for an equilibration phase of 15 min. After 15 min, the NI was recorded, and anesthetic depth was assessed clinically using a score between 1 (“too deep” plane) and 4 (“too light” plane), with a score of 2 reflecting a “medium–deep” anesthetic plane and a score of three a “medium” plane (Table 1). If horses were between two planes based on their clinical parameters, half scores were recorded. For the clinical score adapted from Hubbell and Muir (17), spontaneous movement led automatically to the evaluation as “too light.” For possible major movement, a thiopentone bolus of 5 mg kg^−1^ was prepared for IV injection.

### Statistical Analysis

The Kolmogorov–Smirnov test and histograms were used for evaluation of normal distribution. Correlation was analyzed by Spearman rank correlation coefficient. A *p* < 0.05 was considered significant.

## Results

Vital parameters ranged in clinical acceptable limits for anesthetized horses in all individuals during anesthesia. None of the horses experienced severe hypotension (MAP < 60 mmHg), hypoxemia (PaO_2_ < 10.7 kPa) (<80 mmHg), severe hypercapnia (PaCO_2_ > 8 kPa) (>60 mmHg), hypocapnia (PaCO_2_ < 4 kPa) (<30 mmHg), or hypothermia (rectal temperature <34°C). Therefore, it was not necessary to increase the dobutamine infusion rate in any of the horses. During the study, none of the horses showed movement requiring thiopentone injection.

**Figure 1:**
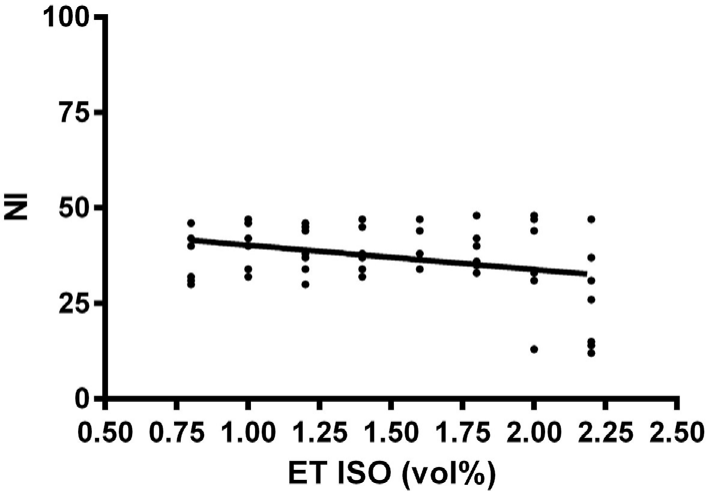
**Relationship of the dimensionless Narcotrend index (NI) with eight different end tidal isoflurane concentrations (ET ISO) in volume percent (vol%) after an equilibration phase of 15 min in seven horses**. The bold line included in the scatter plot is the regression line.

**Figure 2:**
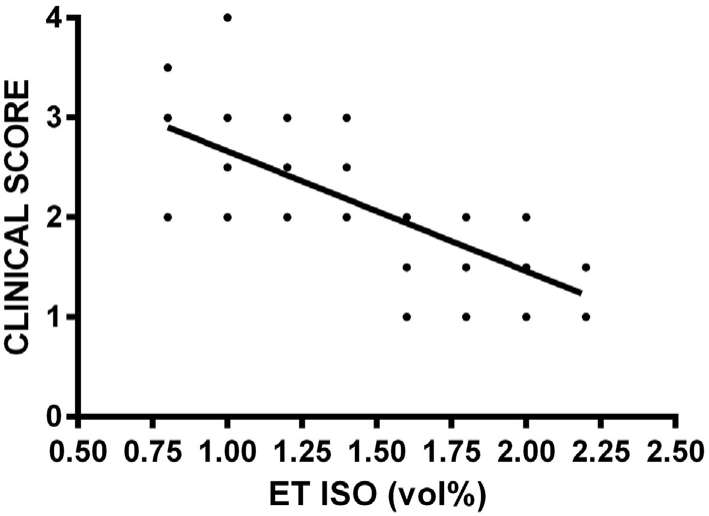
**Relationship of a clinical score between 1 and 4 evaluating anesthetic depth with eight different end tidal isoflurane concentrations (ET ISO) in volume percent (vol%) after an equilibration phase of 15 min in seven horses**. The clinical score is based on common physical signs of anesthetic depth, such as eye reflexes, pupil size and position, respiration, heart rate, and arterial blood pressure [modified from Hubbell and Muir (17)], with a score of 4 reflecting a “too light” anesthetic plane, score of 3 reflecting a “medium” anesthetic plane, score of 2 reflecting a “medium–deep” anesthetic plane, and score of 1 reflecting a “too deep” anesthetic plane. The bold line included in the scatter plot is the regression line.

Over a wide range of lower ET isoflurane concentrations, the NI did not change considerably. Only at high ET concentrations of 2.0 and 2.25 vol%, a sudden decrease of the NI was observed in four of seven horses (Figure 1), and the correlation between NI and isoflurane concentration was poor (*r*_s_ = 0.24; *p* = 0.078). However, the clinical score decreased continuously with increasing isoflurane concentrations (Figure 2), and correlation was good (*r*_s_ = 0.80; *p* < 0.001).

## Discussion

In the present study, NI did not change over a wide range of isoflurane concentrations in all of the seven horses. In contrast to the clinical evaluation, NI did not detect a decrease in anesthetic depth at the lower ET isoflurane concentrations. Only at very high isoflurane concentrations (2.0–2.2 vol%), a decrease in NI was observed.

The results presented here are in accordance with an investigation about a different multivariate EEG monitor, the BIS, in horses (11). In this study, BIS was not able to distinguish between the sedated and the anesthetized state and paradoxically increased at an ET isoflurane concentration of 1.9 vol% when compared to 1.4 vol%. In contrast, in a study in horses receiving halothane or sevoflurane anesthesia, BIS detected a difference in CNS depression between anesthetized and awake horses (19).

One explanation for these conflicting results could be a specific effect of isoflurane on the equine EEG. In a study investigating the effects of volatile anesthetics on EEG spectral parameters, an increase in ET isoflurane concentration from 1.25 to 1.5 MAC paradoxically increased spectral edge frequency of the equine EEG (19). In a different study, isoflurane similarly increased spectral edge frequency, whereas halothane and methoxyflurane reduced it with increasing concentrations (20). In a recent experimental study about the effects of monoanesthetics with different volatile agents on the equine EEG, none of the quantitative EEG data provided useful information to monitor anesthesia and offered little advantage over the use of changes in MAP for this purpose (21). The BIS reversely increased with increasing isoflurane MAC levels in that study (21). However, when propofol was used instead of volatile agents for anesthetic maintenance in horses, BIS was also not different between multiples of the propofol minimum infusion rate (MIR) in anesthesia and correlation with MIR was only moderate (22).

Another possible explanation for the poor correlation in our study is that multivariate monitors, such as Narcotrend, have been developed for use in humans. For example, the Narcotrend incorporates algorithms based on the human sleep EEG, which may not completely be transferable to other species. In dogs anesthetized with isoflurane or sevoflurane, similar to the horses reported here, NI was not different between anesthetic planes assessed clinically as “too light” and as “adequate” but differentiated those planes from a clinical evaluation as “too deep” (15). The difficulty of evaluating the use of such EEG monitors is the lack of some “gold standard” reference method, which determines the hypnotic component of anesthesia precisely. In veterinary anesthesia, commonly used variables include reflex response, muscle tone, respiratory, and hemodynamic parameters, which only indirectly reflect the hypnotic state of a patient.

Clinical evaluation of anesthetic depth seemed to be superior to NI in the examined horses due to better correlation with isoflurane concentrations and differentiation between a “too light” and an “adequate” plane of anesthesia. However, the loss of reflex movement is usually due to inhibition of sensory or motor pathways at the segmental level of spinal cord or brainstem (23, 24), and isoflurane has been shown to inhibit spinal motor neurons and produce immobility primarily by its effect at the spinal cord level (25, 26). Furthermore, it has been shown that the inhalant agent concentration, required to induce unconsciousness and amnesia, is only 20–45% of that required to suppress purposeful movement (27). Consequently, it is possible that the horses reported here did not experience “awareness,” while increase of reflex response and muscle tone were leading to the clinical assumption of a “too light” anesthesia.

Autonomic nervous responses, such as hemodynamic responses, are also commonly used as indicators of anesthetic depth and have been included in the clinical score used in the horses presently reported. However, hemodynamic responses are primarily subcortical in origin and are not depending on corticocerebral input. Therefore, they may not indicate the conscious perception of noxious stimuli (28). Additionally, they are affected by several factors, such as the pharmacodynamic properties of the different anesthetics, the duration of anesthesia, and physiologic factors. Anesthetic concentrations preventing autonomic reflexes and responses are even higher than anesthetic concentrations preventing movement (29). This corroborates the assumption that horses may still have been centrally depressed, while clinical scoring already evaluated anesthetic plane as being “too light.”

The Narcotrend is primarily a monitor of the hypnotic component of anesthesia (30). Therefore, its use in horses might be questionable, because immobility is particularly mandatory for the equine surgeon, and the use of neuromuscular blocking agents is less common than in human anesthesia. However, if reliable monitoring of central depression would be available, the use of drugs with muscle relaxing properties in a multimodal anesthesia approach could reduce doses of general anesthetics and thereby their risk for side effects.

Xylazine slows the EEG in horses (31–33); therefore, the xylazine constant rate infusion used in our horses might have contributed to the fact that the NI did not increase at lower isoflurane concentrations. However, because xylazine was infused at a constant dose over a long infusion period throughout the study, we assume that it causes a continuous background dampening of the EEG. This should not completely mask a dose-dependent effect of isoflurane on the EEG. When investigating EEG effects of centrally acting agents, drug administration is usually restricted to the investigated drug (34). Due to animal welfare, this was not possible in the horses reported here, because they were part of a surgical trial. Furthermore, we wanted to mimic clinical conditions to test the use of the NI to monitor clinical patients. Other drugs included here, as in other EEG studies, were guaiphenesin and thiopentone (35, 36). Guaiphenesin has been shown to have minimal influence on the equine EEG (37), and thiopentone significantly reduced the spectral edge frequency in halothane-anesthetized horses only for 10 min (38).

In four of the seven horses, NI began to decrease at the high ET isoflurane concentrations of 2.0 and 2.2 vol%, respectively, which implicates that NI can distinguish a “too deep” anesthetic plane from an “adequate” plane. Isoflurane is known to suppress the EEG dose dependently and to cause burst suppression even at clinical relevant doses (39, 40). A special algorithm for the recognition of burst suppressions is incorporated in the Narcotrend, which leads to calculation of low indices. Therefore, the decrease of NI could be explained by an increasing amount of burst suppressions or suppression lines at high isoflurane concentrations. The clinical score assessed anesthesia as “too deep” at lower ET isoflurane concentrations, beginning from 1.6 vol%. Therefore, the clinical advantage of NI for recognition of a deep anesthetic plane is questionable.

However, our study is not free of limitations. First, ET isoflurane concentrations used for the correlation were only indicators of effective concentrations, because equilibration phases of 15 min for the different end expiratory isoflurane concentrations were probably too short for equilibration of alveolar anesthetic partial pressure with arterial partial pressure and brain partial pressure. However, differences between the inspiratory and ET isoflurane concentrations were within 10% at the end of the equilibration phases, as recommended by Eger and Bahlman (41). Second, we used a very general clinical score modified from Hubbell and Muir to assess anesthetic depth in horses. This score might not have been ideal for a xylazine balanced inhalation anesthesia, because horses may appear more superficial than they really are, and the palpebral reflex is less dampened in an alpha_2_-agonist balanced inhalation anesthesia (42). Third, clinical usefulness of NI was investigated in a limited number of horses, and the lack of a reference method to measure unconsciousness precisely aggravates the evaluation of EEG monitors.

## Conclusion

Correlation of NI with different ET isoflurane concentrations in horses in balanced anesthesia was poor. Common clinical parameters seemed to be more useful to ensure appropriate surgical conditions.

## Author Contributions

JT contributed to the design of the study, collection and analysis of the data, and prepared the manuscript. KH contributed to the design and collection of data. SK designed the study, analyzed the data, reviewed and finalized the manuscript.

## Conflict of Interest Statement

The authors declare that the research was conducted in the absence of any commercial or financial relationships that could be construed as a potential conflict of interest.
